# Density of fresh wall of acute aortic dissection with synchrotron-based x-ray phase tomography

**DOI:** 10.1093/icvts/ivae157

**Published:** 2024-09-13

**Authors:** Koki Yokawa, Masato Hoshino, Naoto Yagi, Yutaka Nakashima, Kazunori Nakagawa, Yutaka Okita, Kenji Okada, Takuro Tsukube

**Affiliations:** Division of Cardiovascular Surgery, Kobe University Graduate School of Medicine, Kobe, Japan; Research & Utilization Division, Japan Synchrotron Radiation Research Institute/SPring-8, Sayo, Hyogo, Japan; Research & Utilization Division, Japan Synchrotron Radiation Research Institute/SPring-8, Sayo, Hyogo, Japan; Division of Pathology, Japanese Red Cross Fukuoka Hospital, Fukuoka, Japan; Pathophysiological and Experimental Pathology, Graduate School of Medical Sciences, Kyushu University, Fukuoka, Japan; Division of Cardiovascular Surgery, Kobe University Graduate School of Medicine, Kobe, Japan; Division of Cardiovascular Surgery, Takatsuki General Hospital, Takatsuki, Japan; Division of Cardiovascular Surgery, Kobe University Graduate School of Medicine, Kobe, Japan; Division of Cardiovascular Surgery, Kobe University Graduate School of Medicine, Kobe, Japan; Division of Cardiovascular Surgery, Japanese Red Cross Kobe Hospital, Kobe, Japan

**Keywords:** synchrotron-based X-ray phase-contrast tomography, acute aortic dissection, fresh aortic wall, tunica media

## Abstract

**OBJECTIVES:**

The mechanisms behind the onset of acute aortic dissection have not been fully elucidated. We developed dynamic synchrotron-based X-ray phase-contrast tomography to quantitatively study the dynamics of biological samples and applied it to the fresh aortic wall in acute type-A aortic dissection (ATAAD).

**METHODS:**

Fresh, ring-shaped aortas undergoing aortic repair in ATAAD were measured in a container filled with normal cold saline within 24 h of surgery. As a control, we obtained 5 formalin-fixed normal ascending aortas from autopsies (female: 2, 59.7 years) [standard deviation (SD): 5.5 years]. To evaluate the quantitative morphological change, we estimated the density at each step stretched by 2 mm per step. The fresh specimens were analysed pathologically using the area ratio of the elastic fibres.

**RESULTS:**

Samples were obtained from 5 patients [1 man and 4 women, 59.4 (SD: 8.7) years]. The overall density of the tunica media (TM) in the fresh aorta was 1.062 (SD: 0.006) g/cm^3^ and differed significantly between the dissected and non-dissected portions [1.05 (SD: 0.004) vs 1.066 (SD: 0.004) g/cm^3^, respectively; *P* = 0.0122]. When the fresh aortic wall was stretched and became thinner, the density of the TM remained unchanged. Compared with the pathological findings, the area ratios of the elastic fibres of the TM were lower in the non-dissected portion than normal [48.6 (SD: 7.1)% vs 60.5 (SD: 5.7 %, *P* < 0.001].

**CONCLUSIONS:**

Dynamic synchrotron-based X-ray phase-contrast tomography can trace the deformation process that occurs in situ in fresh aorta in ATAAD. We confirmed that the densitometric property of the aortic wall in ATAAD was unchanged during the stretching process.

## Graphical abstract

**Figure:**
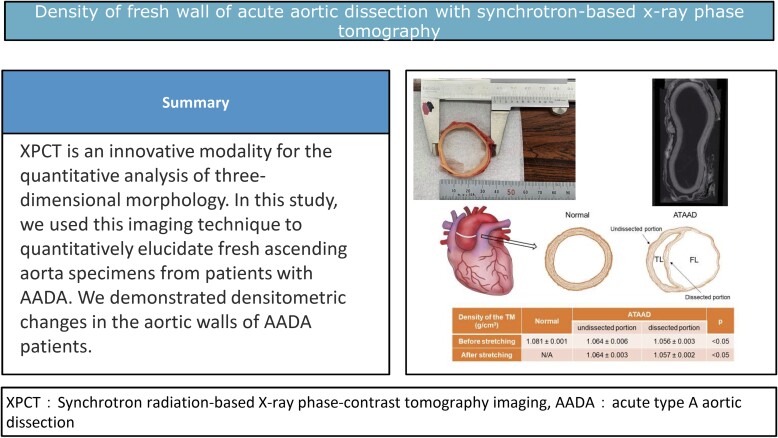

## INTRODUCTION

Synchrotron radiation (SR) is inherently suited to the dynamic imaging of small areas [1, 2]. The high sensitivity of phase-contrast imaging facilitates the visualization of small density differences in biological soft tissue, which often represent important structural information. We have previously shown that SR-based X-ray phase-contrast tomography (XPCT) exhibited differences in the structure of the aortic wall in acute type-A aortic dissection (ATAAD) specimens from patients with and without Marfan syndrome (MFS) and in normal aortas [3]. Medial density was homogeneous in the normal aortas, markedly varied in those with MFS and significantly lower and different among those without MFS. These changes may be present in the tunica media (TM) before the onset of aortic dissection. The application of XPCT to obtain densitometric data for aortic diseases could be useful for investigating their pathogenesis [4–6].

It is also important to understand morphological changes to the aorta under loaded conditions to clarify the mechanisms underlying aortic diseases. Previously, we developed a technique for the quantitative dynamic X-ray phase-contrast imaging of nonfixed fresh biological samples, based on intermittent stretching (dynamic XPCT; *d*-XPCT) that simulates in situ conditions [5].

The objective of the present study was to apply *d*-XPCT to fresh ATAAD aortic walls to observe structural changes under stretching.

## MATERIALS AND METHODS

### Ethical statement

This study was approved by the institutional review board of the Japanese Red Cross Kobe Hospital (No. 26, 30 January 2013), and informed consent was obtained from all patients. The investigation conformed to the principles in the Declaration of Helsinki. Fresh normal human aortas were not used; a formalin-fixed human aorta was used only for histological analysis because the institutional review board did not approve the use of fresh normal human aortas in this study.

### Aorta specimens

Rings from the dissected ascending aortas were freshly obtained from patients with AADA (*n* = 5; 1 man and 4 women) who underwent emergency aortic replacement at the Japanese Red Cross Kobe Hospital. Each specimen was an approximately 1-cm wide transverse aortic ring collected 3 cm above the sinotubular junction of the ascending aorta. The fresh aortas were immediately stored in cold saline at 4°C and subjected to *d*-XPCT within 24 h.

### X-ray phase-contrast tomography imaging

**Figure 1:**
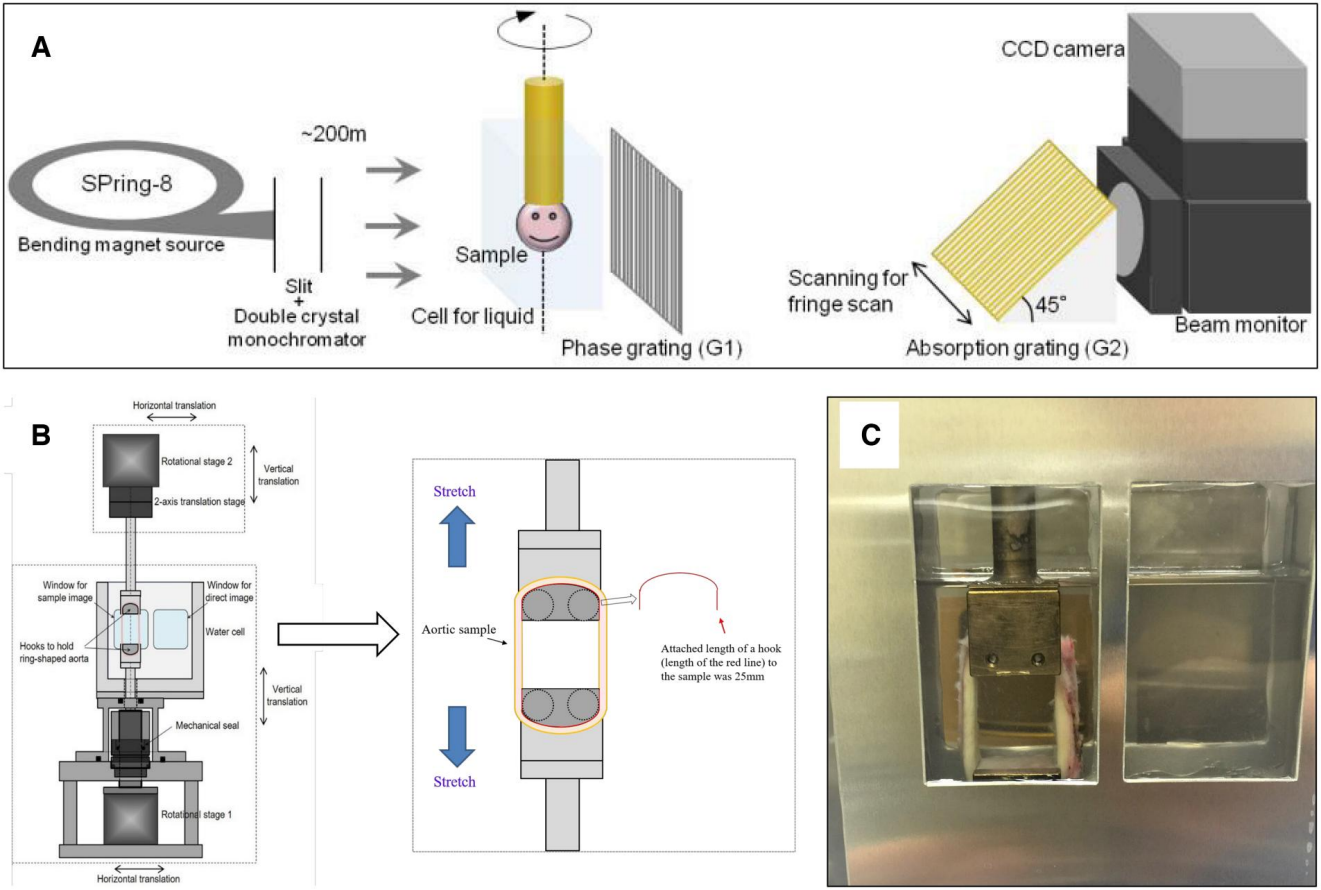
Schematic drawing of the experimental set-up for the dynamic X-ray phase-contrast imaging based on the grating interferometer. (**A**) Talbot grating interferometer, consisting of a phase grating and an absorption grating, was placed behind the specimen. (**B**) Schematic drawing of X-ray phase contrast tomography for dynamic measurement of fresh aorta under stretching conditions. (**C**) Photograph of a ring-shaped fresh human ascending aorta of acute aortic dissection set on a sample stage. CCD: charged couple device.

The XPCT system in the SPring-8 SR facility, which is based on an X-ray Talbot grating interferometer in the bending-magnet synchrotron beamline (BL20B2), has been described in detail elsewhere [3, 5, 7]. Briefly, the system was located 200 metres from an X-ray source, and tuned to 25 keV by passing it through a silicon double-crystal monochromator (Fig. 1A). The data were processed by a high-throughput system to create 3-dimensional images. The specifications of the system were as follows: field of view, 24.7 mm (H) × 17.1 mm (V); voxel size, 12.5 μm; target density range, 0.9–1.2 g/cm^3^; density resolution, 1 mg/cm^3^; exposure time, 1 s/image; and number of projections, 900. The horizontal field of view could be extended up to 40 mm by employing an offset tomographic scan. A physical quantity that is about 1000 times larger than that in the attenuation is involved in the phase shift; thus, in principle, X-ray phase tomography has a high sensitivity for soft tissues [2, 3, 7]. The ability to visualize small differences in density and fine details in the structure is attributable to high spatial and density resolution [4].

The reconstructed volumes were converted into 16-bit TIFF images of each tomography slice with custom-made original software. Further image processing and analysis of the tomography data were performed with ImageJ (http://rsbweb.nih.gov/ij/index.html) [3, 5, 7]. Morphological differences were quantitatively evaluated by estimating the mean mass density of the TM within 8 regions of interest in the aortic wall for each formalin-fixed sample. Each region of interest was 80 × 80 × 10 voxels.

### Dynamic X-ray phase-contrast tomography

The aorta is constantly exposed to blood pressure and subjected to physical loads. As a result, understanding morphological changes to the aorta under loaded conditions may be useful for elucidating the mechanisms underlying diseases such as ATAAD. However, it is difficult to use XPCT to observe the aorta in vivo with high image contrast because bones and air bubbles in the body produce excess phase shifts at their boundaries, making it difficult to retrieve accurate phase information.

As an alternative to in vivo measurement, a dynamic measurement system has been developed that uses XPCT to measure the properties of an extracted aorta in a stretched condition that emulates in situ conditions. The system has been described in detail elsewhere [5]. Briefly, a sample stage was modified to hold and stretch a ring-shaped aorta (Fig. 1B). Driving the 2 rotational stages synchronously allows the sample to be rotated in its stretched condition. Ring-shaped fresh ascending aorta specimens were trimmed so that the width of the ring was approximately 10 mm (Fig. 1B), and these were put in saline and kept in a refrigerator prior to measurement. The diameter of the rings without any pressure was approximately 35 mm. For the measurements, a 25-mm-deep water cell was filled with normal saline, and metal hooks were attached to the rotating shafts to hold the ring-shaped aorta specimen in place. These metal hooks were elliptically shaped and were attached to the samples along a length of 25 mm each. Metal hooks were attached to the rotating shafts to hold the ring-shaped aorta. Figure 1C shows the ring-shaped fresh aorta set on the sample stage. Tension was applied to the aorta in the initial state to prevent it from dropping from the hooks during measurement. To demonstrate a dynamic measurement, hooks were used to stretch the aorta by increments. The scope of the stretch was 2 mm per step (1 mm by the upper hook and 1 mm by the lower hook). The stretching and tension (N) applied to each specimen were monitored simultaneously. The measurements were taken under the following conditions: number of projections, 600, with five-step phase stepping; exposure time, 500 ms per image; and voxel size, 15.5 μm. Images were taken of the middle part of the aorta located between the hooks. The XPCT images of the aorta specimen at its initial diameter and during the stretching phase were analysed, and measurements were taken of the density of the TM for each step. On the image, the mean value was estimated from the region of interest of 8 sections of 40 × 40 × 10 voxels for the smaller region, or 80 × 80 × 10 voxels for the larger region, The region of interest for the measurement was set as closely as possible to the same position, but with different degrees of stretching. In comparison with XPCT imaging and histological examinations, the mean value was estimated from the region of interest of a section of 80 × 80 × 1 voxels for the pathological sections.

### Histological examinations

After completion of the XPCT imaging, the samples were fixed in 10% formalin and sectioned transversely. Thereafter, sections of each specimen were stained with haematoxylin and eosin, elastica van Gieson (EVG), Masson's trichrome (MT) and Alcian blue stains [3]. The EVG, MT and Alcian blue stains were used to detect elastic fibres, collagen fibres and acid mucopolysaccharide, respectively. Smooth muscle cells (SMCs) were identified by immunostaining with anti-α-smooth muscle actin (α-SMA) antibody (DAKO, Denmark A/S, Glostrup, Denmark). The histological examination focused on the structural changes of the elastic fibres, laminar medial necrosis, atherosclerosis, fibrosis of the media and any changes to the vasa vasorum. Laminar medial necrosis was defined as laminar or band-like loss of the media’s SMCs [8].

Moreover, to assess the contribution of elastic fibres to the XPCT image, the area ratio of the aortic components, such as elastic fibres, collagen fibres and SMCs, was observed in all samples of aortic dissection and the controls. In a section of each specimen stained with EVG, an area 1000 × 1000 mm square was selected in the TM. Then, the area ratio of the elastic fibres in each area was analysed using Adobe Photoshop (San Jose, CA, USA) by an experienced pathologist (Y.N.) and compared to the mass density of the same area in the XPCT image.

### Statistical analyses

The statistical tests were performed with JMP statistical software (SAS Institute, Cary, NC, USA). Data are expressed as mean (SD). The Kruskal-Wallis test was used to compare the densities of the aortas in the TMs and the area ratio of elastic fibres between non-dissected portion and control samples. In the comparison between the dissected and non-dissected regions, we used the Wilcoxon test for analysis. In the comparison using d-XPCT, we employed a linear mixed model to analyse changes in density. Data are expressed as mean (SD). For the analysis of density and elastic fibres, we used linear regression. For the relationship between the average density measurements and the densities in other extracted sections, we utilized correlation regression and indicated the value of Spearman's *r*.

## RESULTS

### Patient characteristics

The mean age of the patients with ATAAD was 59.4 (SD: 8.7) years. Patients with MFS were excluded. Patient characteristics are shown in Table 1. Four patients were diagnosed with hypertension and used oral antihypertensive medication preoperatively, including an angiotensin receptor blocker (*n* = 2), a calcium channel blocker (*n* = 1), a beta-blocker (*n* = 1) and an alpha-blocker (*n* = 1). In addition, 5 formalin-fixed normal ascending aortas were obtained from autopsies [2 from women; the mean age was 59.7 (SD: 5.5) years]. CT findings are also shown in Table 1. The primary entry was located in the ascending aorta (*n* = 3) and the aortic arch (*n* = 2). All of the false lumens were patent, and the diameter of the ascending aorta was 45 (SD: 5.4) mm.

**Table 1:** Characteristics of patients who supplied the fresh acute type A aortic dissections

	ATAAD
Numbers of patients	5
Age (years)	59.4 ± 8.7
Sex (male)	1
Hypertension	4
Anti-hypertensive medication	
Calcium channel blocker	1
β-Blocker	1
Angiotensin receptor blocker	2
α-Blocker	1
Dyslipidaemia	1
Coronary arterial disease	2
Chronic kidney disease	1
**CT findings**	
Location of intimal tear	
Ascending aorta	3
Aortic arch	2
Ascending aorta diameter (mm)	42 ± 5.4
Patent false lumen	5
**Procedures**	
Total arch replacement	2
Hemi-arch replacement	3

ATAAD: acute type-A aortic dissection; CT: computed tomography.

### Dynamic assessment of the tunica media of the fresh aorta

In the fresh samples of dissecting aortas (*n* = 5), the mean density of the TM in non-dissected portions of the aortic wall was 1.066 (SD: 0.004) g/cm^3^ (range, 1.059–1.073 g/cm^3^) before stretching the aortic wall. In addition, the mean density of the TM in the dissected portions was significantly lower than that of the non-dissected portions [1.057 (SD: 0.004) vs 1.066 (SD: 0.004) g/cm^3^, respectively; *P* = 0.0122].

**Figure 2:**
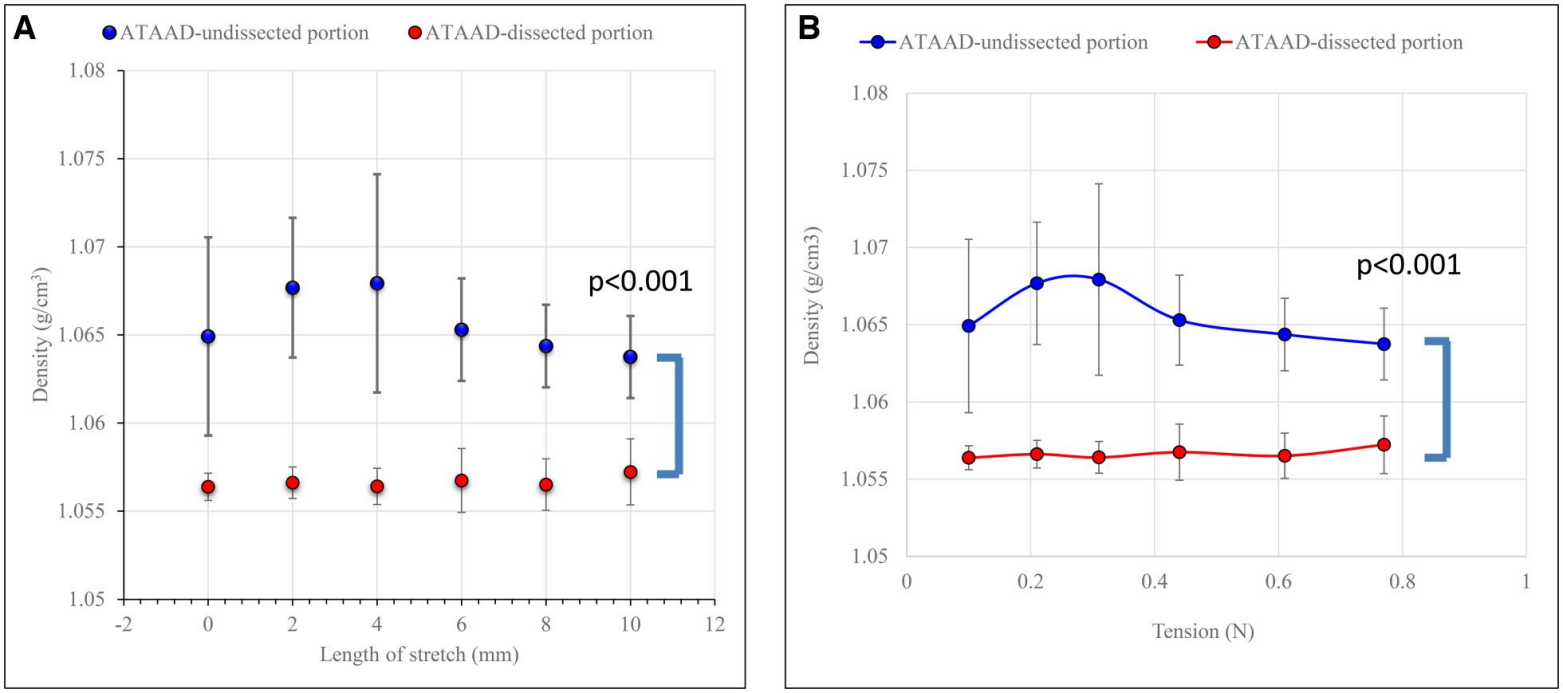
Changes in the density of the tunica media of fresh aortas during stretching. (**A**) Relationship between the density of the tunica media and the length of stretching of the non-dissected portion of the acute type A aortic dissection (blue) and the dissected portion of the acute type A aortic dissection (red). (**B**) Relationship between the density of the tunica media and wall tension during stretching of the non-dissected portion of the acute type A aortic dissection (blue) and the dissected portion of the acute type A aortic dissection (red). ATAAD: acute type-A aortic dissection.

Dynamic XPCT was applied to all the fresh samples (Fig. 2A and B). The mean diameter of the aortic ring was initially 34.7 (SD: 1.5) mm and the increased ratio was 29.1 (SD: 1.3) % after stretching by 10 mm (Fig. 2A). The wall thickness of the non-dissected portion of the aorta was initially 2.92 (SD: 0.16) mm, decreasing to 2.57 (SD: 0.15) mm at the end of the stretching process. There was a 12.1% reduction in wall thickness. The initial width of the non-dissected portion of the aorta was 8.21 (SD: 0.14) mm, decreasing to 7.60 (SD: 0.07) mm at the end of the stretching process. There was a 7.6% reduction in the width of the aortic wall. The tension of the aortic wall was initially 0.10 N, increasing to 0.77 (SD: 0.02) N at the end of the stretching process (Fig. 2B). The density of the TM of the same region did not increase under tensile force; it was 1.064 (SD: 0.003) g/cm^3^ (range, 1.060–1.067 g/cm^3^) after stretching by 10 mm. Similarly, the density of the TM of the dissected portion was 1.057 (SD: 0.002) g/cm^3^ after stretching by 10 mm and did not increase under tensile force (Fig. 2A and B). There was a significant difference between these groups (*P* < 0.001).

### Comparison with pathological findings

**Figure 3:**
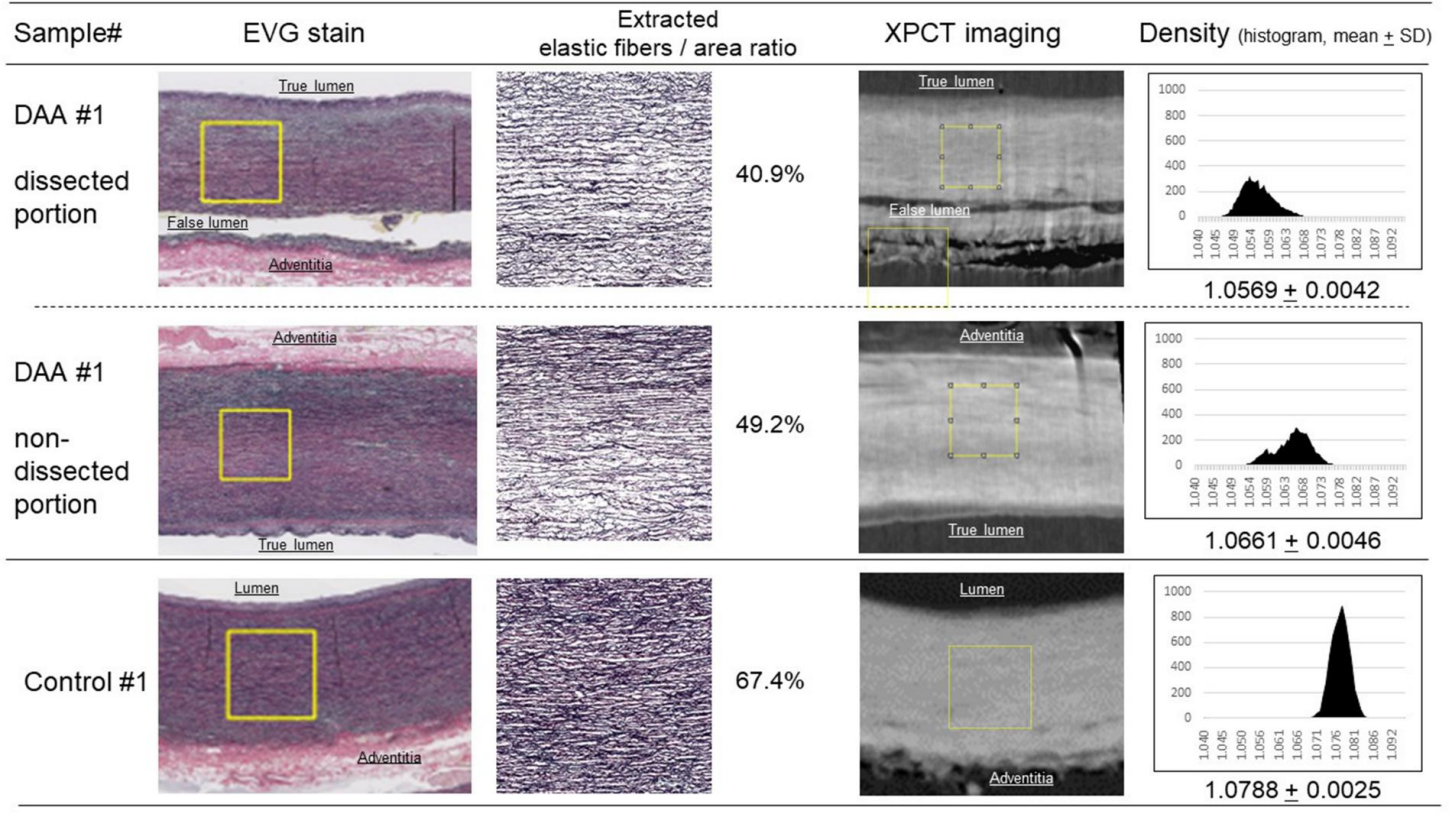
Representative histological findings (Elastica van Gieson stain and area ratio of elastic fibres), X-ray phase-contrast tomography imaging and density (histogram and mean + SD) in the same measurement area. (Upper) Dissected portion of the acute type A aortic dissection #1 sample. (Middle) Non-dissected portion of the acute type A aortic dissection #1 sample. (Lower) Aortic wall of the control #1 sample. Yellow square (1 × 1 mm square) describes the measurement area.

**Figure 4:**
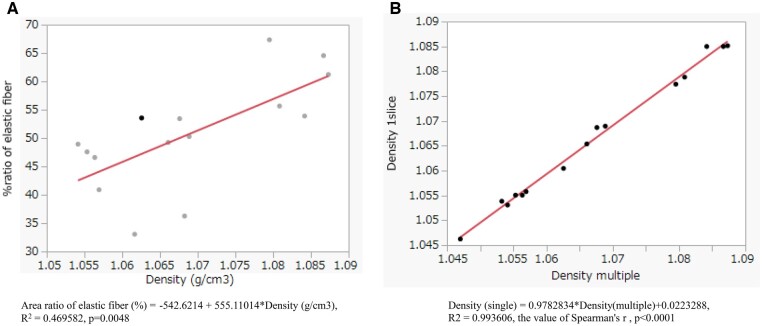
(**A**) Correlation between the area ratio of the elastic fibres (%) and the density of the tunica media (g/cm^3^). (**B**) Correlation between the mean tissue density of the tunica media estimated by multiple sections (8 sections of 80 × 80 × 10 voxels) and a single section (1 section of 80 × 80 × 1 voxels).

To quantitatively evaluate the morphological differences, the area ratio of the elastic fibres and the densities of the TM were estimated and compared with those of normal aortas. Figure 3 shows a comparison of the EVG stain, the area ratio of the elastic fibre (%), an XPCT image and the distribution and mean of the density of the same measurement area of the dissected portion of the ATAAD aorta, the non-dissected portion of the ATAAD aorta and a normal aorta. Density variations in the TM were consistent with the lower area ratio of the elastic fibres in the ATAAD aortas, including both the dissected portion and the non-dissected portion, and the area ratio of elastic fibres in the dissected portions was lower than that of the non-dissected portions. In contrast, distribution of the elastic fibres and the density of the media of the normal aorta were homogeneous, providing the vessels with a stable structure. Table 2 summarized the area ratio of the elastic fibres and the density of the TM of the measurement area of all the samples. Overall, there was significant correlation between the area ratio of the elastic fibres and the density of the TM (Fig. 4A). In the dissected portion of the ATAAD aorta, the area ratio of the elastic fibres and the density of the TM were 43.4 (SD: 6.6) % and 1.054 (SD: 0.004) g/cm^3^, respectively and were significantly lower compared to those of the non-dissected portion of the ATAAD aorta [(48.6; SD: 7.1) % and 1.064 (SD: 0.006) g/cm^3^, respectively, *P* = 0.0088)]. In normal aorta, the area ratio of the elastic fibres and the density of the TM were 60.5 (SD: 5.7) % and 1.084 (SD: 0.003) g/cm^3^, respectively and were significantly higher compared to those of the non-dissected portion of the ATAAD aorta (*P* = 0.012). As Fig. 4B demonstrates, the mean density of the measurement area of a 1000 mm square with a depth of 12.5 mm strongly correlated with the mean density of the multiple measurement area; for 8 sections of 1000 mm square, each with 100 mm depth (*P* < 0.001), the densitometric changes of the TM might be consistent through all of the samples.

**Table 2:** Area ratio of the elastic fibres (%) and tissue density of the tunica media (g/cm^3^) in each sample

Group	Sample #	Ef area ratio	Density		
Control	1	67.4	1.077		
Control	2	55.7	1.079		
Control	3	61.2	1.085		
Control	4	53.9	1.085		
Control	5	64.6	1.085		

Group	Sample #	Non-dissected portion		Dissected portion	
		Ef area ratio	Density	Ef area ratio	Density
ATAAD	1	49.2	1.066	40.9	1.057
ATAAD	2	36.3	1.069	33.1	1.063
ATAAD	3	50.3	1.069	48.9	1.054
ATAAD	4	53.4	1.068	47.6	1.055
ATAAD	5	53.6	1.063	46.6	1.056

ATAAD: acute type-A aortic dissection; Ef: elastic fibre.

## DISCUSSION

We have previously demonstrated that SR-based XPCT is an innovative modality for the analysis of 3-dimensional morphology and is useful for understanding the pathophysiology of aortic dissection [3]. In this study, we made a quantitative comparison between several types of aortic walls, using formalin-fixed samples, which revealed differences in the microstructure of the aortic wall in specimens from patients with aortic dissection and those in specimens from normal aortas. The tissue density in the TM of normal ascending aortas was almost homogeneous, with a single peak in the density histogram at approximately 1.080 g/cm^3^. In contrast, the aortas of patients with ATAAD without MFS showed a significantly lower density of the TM (1.066 ± 0.003 g/cm^3^). In addition, the density in the XPCT image appeared to be determined mainly by the elastic fibres in the TM in the aortic dissection [3]. These differences in the densitometrical changes of the TM correspond to differences in pathological findings reported in previous studies [9, 10]. Importantly, under unloaded conditions, the tissue density in the TM of fresh ATAAD aortas used in the present study coincided with the densities of the ATAAD aortas in our previous study [3].

The pathological findings from previous studies were obtained using formalin-fixed samples under unloaded conditions [8–10]. However, under physiological conditions, the aortic wall is exposed to blood pressure and subjected to physical loads, which enlarges its diameter and reduces the thickness of the aortic wall [11, 12]. To emulate in situ conditions, we developed a dynamic XPCT system and applied it to extracted fresh aortas [5]. In this study, we first described structural alterations in the aortic wall when stretched. When the ring-shaped aortic specimens were stretched, the wall became thinner, and significant differences in the mass density of the stretched TM reappeared. In fresh ATAAD aortas, no remarkable increase in density was observed along both the non-dissected and the dissected portions in a stretched condition. This difference between ATAAD aortas and normal aortas could be explained by variations in aortic components, such as elastic fibres, collagen fibres, acid mucopolysaccharide (including glycosaminoglycans) and SMCs [13–15]. Because elastic fibres, collagen and microfibrils of the connective tissue and their interactions with the medial SMCs are critical for connective tissue contraction and important for maintaining high interstitial fluid pressure [15, 16], the decrease in elastic fibres may lose the low rebounding resilience of a fresh aortic wall against stretching.

The microstructure of dissecting aortas is characterized by a loss of elastin content, a decrease in interlaminar elastic fibre and a decrease in collagen concentration, especially from the inner media to the outer half [17]. Mallat *et al.* [16] proposed that alterations in microvascular tone and interstitial pressure generation would induce interstitial oedema and a swelling of osmotically active glycosaminoglycans, particularly in the presence of altered connective tissue [18, 19]. This situation would alter stress transfer and generate significant heterogeneities in stress concentration within the aortic wall, potentially initiating a vicious circle. Multiple intraparietal oedemas eventually coalesce, leading to intraparietal dissection, while reduced contractile force in medial SMCs, coupled with the alteration of structural components of the extracellular matrix, limits extracellular matrix contraction, further promoting the formation of intramural oedema—a critical step in the initiation of aortic dissection [16]. Their concept is strongly supported by densitometric observations in TM of the ATAAD aortas in this study. In addition, regarding the portion of the aorta that we estimated, the proximal ascending aorta, Ramaekers *et al.* reported the characterization of ascending aortic flow in patients with degenerative aneurysms using 4-dimensional flow magnetic resonance imaging [20]. These researchers revealed a decrease in flow vorticity and helicity and an unequal distribution of wall shear stress (WSS), with a higher peak WSS in patients with thoracic aortic aneurysm, especially the proximal ascending aorta that we elucidated. High WSS and positive WSS gradients have been shown to result in adapted gene expression in endothelial cells, which initiates endothelial proliferation, extracellular matrix degradation and aneurysm formation. Our current study also showed the degeneration of the ascending aorta with acute aortic dissection. The SR-based XPCT could be one of the imaging modalities that accurately detect changes in aortic components and interstitial oedema in predicting aortic disease progression and susceptibility to dissection. In addition, although the densities of the TM of the ATAAD were lower than those in the normal aorta, higher densities of the TM along the non-dissected portion of the ATAAD, when compared with the densities along the dissected portion, could indicate that the medial dissection was terminated at the non-dissected portion in the aortic wall.

Finally, our study demonstrated densitometric changes in the aortic walls of patients with ATAAD. These changes should be useful in identifying vulnerable aortas and potentially in predicting ATAAD. Other strategies for observing densitometric changes may have clinical implications for the development and use of new diagnostic technologies. For example, the development of higher-resolution ultrasonography might be required for the in vivo diagnosis of aortic wall disease, and novel aortic ultrasound imaging would demonstrate the densitometric changes [21, 22].

This study has certain limitations: (i) The density in the XPCT image includes all of the aortic components. Elastin and collagen are key fibrous proteins that are found in the arterial walls. Findings from a representative sample of patients with chronic aortic dissection in our previous study suggested that mass density in the XPCT image in the TM in aortic dissection may be mainly determined by elastic fibres [3]. However, applying those analyses to the samples of acute aortic dissection was challenging. (ii) The sample size, which was 5, was relatively small. The major reason for the small number of sample cases was our study design. Because each schedule for the beamline for the XPCT study was decided 6 months before the measurement, we always had difficulty obtaining fresh aortic samples from the emergency open aortic repairs for ATAAD on the day of the study. Therefore, the number of fresh aortic samples was limited. However, the XPCT analysis was conducted rigorously, with 5 aortic sections selected in both non-dissected and dissected portions per stretch, with a total of 624 regions analysed. (iii) The patients were limited to those younger than 70 years. Because calcified samples are not suitable for XPCT imaging, specimens from older patients were not investigated. (iv) The use of fresh human aortas was not approved. (v) All of the samples were collected from the same area of the ascending aorta, so further studies may be needed to examine the aorta at varying sites. (vi) The technique we use does not allow inferences on other—potentially more relevant—contributing factors for aortic dissection. Impaired elasticity is likely not the only causative factor. Consequently, more extensive research covering different fields would seem necessary to obtain a clearer and broader picture of the underlying pathophysiology.

## CONCLUSION

The ability of the dynamic XPCT to trace the details of the deformation process that simulates the in situ conditions of the biological soft tissues meant that fresh aortas in a state of ATAAD could be analysed during stretching. The structure of the aortic wall differed significantly between ATAAD and normal samples, and the mechanical properties of the aortic wall during stretching differed from those of intact aortas.

## Data Availability

All relevant data are within the manuscript.

## ABBREVIATIONS

ATAAD: Acute type-A aortic dissection
EVG: Elastica van Gieson
MFS: Marfan syndrome
SD: Standard deviation
SMC: Smooth muscle cells
SR: Synchrotron radiation
TM: Tunica media
WSS: Wall shear stress
XPCT: X-ray phase-contrast tomography
